# Supplementary material for: SARS Coronavirus-2 Microneutralisation and Commercial Serological Assays Correlated Closely for Some but Not All Enzyme Immunoassays
Source: Viruses. 2021 Feb 4;13(2):247. doi: 10.3390/v13020247 (PMC7915197; doi:10.3390/v13020247)
Supplement: Supplementary file 1 [file viruses-13-00247-s001.pdf]

Supplementary Materials

SARS Coronavirus-2 microneutralisation and commercial serological assays correlated closely for some but not all enzyme immunoassays

Gregory J Walker, Zin Naing, Alberto Ospina Stella, Malinna Yeang, Joanna Caguicla, Vidiya Ramachandran, Sonia R Isaacs, David Agapiou, Rowena A Bull, Sacha Stelzer-Braid, James Daly, Iain B Gosbell, Veronica C Hoad, David O Irving, Joanne M Pink, Stuart Turville, Anthony D Kelleher, William D Rawlinson

Corresponding author: Prof William Rawlinson, Virology Research Laboratory, Level 3 Clinical Sciences Building, Prince of Wales Hospital, Randwick 2031, Australia.

E-mail address: w.rawlinson@unsw.edu.au

This file includes:

Supplementary method. SARS-CoV-2 spike RBD protein production

Table S1. Samples used to assess sensitivity and specificity of serological assays for SARS-CoV-2

SARS-CoV-2 spike RBD protein production

SARS-CoV-2 Spike RBD was cloned into pCAGGS as described previously (1). The plasmid was transiently transfected into Expi293-Freestyle cells (ThermoFisher Scientific) as follows: 1·5x10^8 total cells (50mL transfection) were mixed with 50 µg of plasmid, 160 µL of ExpiFectinamine and 6 mL of OptiMEM-I and left overnight at 37°C in a shaking incubator. The following day 300 µL of ExpiFectamine Enhancer 1 and 3 mL of ExpiFectamine Enhancer 2 was added to the cells before the cells were left in culture for a further 48 hours. After a total of 72 hours in culture, the cell culture is collected and centrifuged for 20 minutes at 4000xg, 4°C. Cellular debris was clarified by passing the supernatant twice through a 0·22 µM filter. The His-tagged protein was then affinity purified from the cell supernatant using a HisTrap HP Column (GE Healthcare) and eluted with imidazole. The purified protein was then buffer exchanged and concentrated in sterile DPBS by centrifuging at 4000xg for 30 minutes at 4°C in a 10,000 MWCO Vivaspin centrifugal concentrator (Sartorius) and stored at – 80°C. The recombinant RBD was biotinylated using a Biotin Protein Labeling Kit (Roche). To prepare antigen-coated wells for the in-house ELISA, 8-well strips were initially coated with 100µL/well streptavidin (10µg/mL) and then blocked with 5% BSA in TBST. This was followed by coating with biotinylated RBD antigen (100µL/well, 10µg/mL in 0·5% BSA).

Table S1. Samples used to assess sensitivity and specificity of serological assays for SARS-CoV-2

Measurement	Confirmed Infection	Samples
Sensitivity	SARS-coronavirus-2	200
Specificity	N/A	75
	Influenza virus A	7
	Influenza virus B	7
	Enterovirus	5
	Respiratory Syncytial Virus	3
	Adenovirus	1
	Parainfluenza virus type 1	1
	Parainfluenza virus type 3	1

References

1. Amanat F, Stadlbauer D, Strohmeier S, et al. A serological assay to detect SARS-CoV-2 seroconversion in humans. Nat Med. 2020;26(7):1033-6.
